# Screening for undiagnosed obstructive sleep apnea in patients with acute stroke or TIA

**DOI:** 10.1186/s12883-026-05083-1

**Published:** 2026-06-17

**Authors:** Einar Thokle Hovden, Wilhelm Eyde Kielland, Synne Bruun, Simon Holmvik, Fredrik Ildstad, Titto Idicula, Maryam Shirzadi

**Affiliations:** 1 Faculty of Medicine, Norwegian University of Science and Technology (NTNU), Trondheim, Norway; 2 Departement of Internal Medicin, St.Olavs Hospital, Orkdal, Norway; 3 Department of Neuromedicine and Movement Science, Faculty of Medicine and Health Sciences, Norwegian University of Science and Technology (NTNU), Trondheim, Norway; 4 Department of Medicine, Stroke Unit, St. Olavs Hospital, Trondheim, Norway; 5 Department of Neurology and Clinical Neurophysiology, St Olavs Hospital, Trondheim, Norway; 6 Department of Neuromedicine and Movement Science, Norwegian University of Science and Technology (NTNU), Trondheim, Norway

**Keywords:** Obstructive sleep apnea, Ischemic stroke, Transient ischemic attack, STOP-Bang questionnaire, Epworth Sleepiness Scale, Stroke prevention

## Abstract

**Background:**

Obstructive sleep apnea (OSA) is a prevalent and potentially modifiable risk factor for stroke but remains underdiagnosed in clinical practice. Simple screening tools such as the STOP-Bang questionnaire and the Epworth Sleepiness Scale (ESS) may facilitate identification of individuals at risk. However, data on pre-stroke OSA-related symptoms and the utility of these screening tools in acute stroke populations remain limited.

**Methods:**

In this prospective observational study, 124 patients admitted with acute ischemic stroke or transient ischemic attack (TIA) at St. Olavs Hospital, Norway, between April 2023 and January 2026 were included. Excessive daytime sleepiness and risk of OSA prior to the index event were assessed retrospectively using the ESS and the STOP-Bang questionnaire during hospitalization, with most patients evaluated within the first three days after admission. Additional information about risk factors and stroke etiology was extracted from medical records.

**Results:**

The mean STOP-Bang score was 3.2, and 65.3% of patients were classified as having intermediate or high risk of OSA. The mean ESS score was 6.5, with 13.7% meeting criteria for excessive daytime sleepiness (ESS ≥ 11). In adjusted regression analyses, higher STOP-Bang scores were associated with higher ESS scores (β = 0.67, *p* = 0.004), and most patients with elevated STOP-Bang scores did not report excessive daytime sleepiness.

**Conclusions:**

A substantial proportion of patients with ischemic stroke or TIA have a previously unrecognized risk of OSA, while excessive daytime sleepiness is relatively uncommon. STOP-Bang and ESS appear to capture different aspects of sleep-disordered breathing and sleep-related symptoms. STOP-Bang identified a larger proportion of patients at risk of OSA, whereas ESS provided complementary information on subjective daytime sleepiness. Routine screening for OSA symptoms may improve identification of modifiable risk factors relevant for stroke prevention.

## Introduction

Ischemic stroke represents a major global cause of mortality and long-term disability, with an increasing overall burden [1]. Obstructive sleep apnea (OSA) is a prevalent and potentially modifiable risk factor for cerebrovascular disease [2, 3]. These respiratory events lead to intermittent hypoxia, sleep fragmentation, and sympathetic activation, resulting in transient increases in blood pressure and heart rate [4]. Epidemiological studies identify sleep-disordered breathing as an independent risk factor for hypertension, platelet activation, endothelial dysfunction, and early atherosclerosis, thereby increasing the risk of cardiovascular events, including stroke [5–9]. Although OSA is frequently reported among patients with stroke, prevalence estimates vary, and systematic screening in the acute stroke setting is still not performed [10].

Diagnosis of OSA requires polysomnography, which is resource-intensive and often impractical during acute hospitalization. Brief screening tools such as the Epworth Sleepiness Scale (ESS) and the STOP-Bang questionnaire provide simple methods for assessing excessive daytime sleepiness and symptoms suggestive of OSA [11, 12]. Studies show that over 60% of ischemic stroke patients have OSA (apnea-hypopnea index ≥ 10) [13, 14]. However, the burden of these symptoms prior to stroke onset and the clinical utility of these instruments for stroke prevention remain insufficiently characterized.

The primary aim of this study was to determine the pre-stroke or pre-transient ischemic attack (TIA) prevalence of excessive daytime sleepiness and symptoms suggestive of OSA among patients admitted with acute ischemic stroke or TIA. The secondary aim was to examine the utility of STOP-Bang and ESS in these patient groups, to assess the association between ESS and STOP-Bang scores and determine whether these instruments provide complementary information in this clinical setting. Improved identification of OSA may lead to better primary stroke prevention.

## Methods

### Materials

In this observational study, patients were prospectively enrolled at the stroke unit of St. Olavs Hospital, Norway, between April 2023 and January 2026. A total of 124 patients met the eligibility criteria and were included in the study.

Eligible participants were adults (≥ 18 years) admitted with acute ischemic stroke or TIA confirmed by CT or MRI, and with a premorbid modified Rankin Scale (mRS) score < 3 [15]. Patients were excluded if they lacked capacity to provide informed consent, had severe aphasia preventing participation, had documented cognitive impairment prior to the index event, had known OSA or prior use of continuous positive airway pressure (CPAP), or had a history of intracerebral hemorrhage.

Potential participants were identified by the responsible nurse and assessed for eligibility by the study team. All inclusion and exclusion criteria were verified through patient interviews and review of the electronic medical records.

Clinical information, including stroke characteristics, National Institutes of Health Stroke Scale (NIHSS) [16], acute reperfusion treatment, and premorbid functional status, was obtained from the medical records. Information on risk factors, employment status and lifestyle factors was collected through structured patient interviews and cross-checked against available medical documentation when applicable.

Excessive daytime sleepiness and risk of OSA were assessed using the ESS [17] and the STOP-Bang questionnaire [12], administered through structured interviews. The ESS evaluates the participant’s usual level of daytime sleepiness in recent times, whereas the STOP-Bang questionnaire assesses current risk factors and characteristics associated with OSA. Participants were instructed to base their responses on their habitual symptoms prior to the stroke or TIA. ESS is a validated 8-item questionnaire, with total scores ranging from 0 to 24, where higher scores indicate greater daytime sleepiness [17]. The STOP-Bang questionnaire and scoring criteria are presented in Table 1. A Norwegian version of the STOP-Bang questionnaire was developed and validated using a back-translation procedure. The ESS was already available in a validated Norwegian version [17]. Assessments were conducted during hospitalization, primarily within the first three days after admission for the majority of patients.

Participants with incomplete questionnaire responses were contacted by telephone to obtain the missing information when possible.

NIHSS score, mRS, ESS and STOP-Bang are all commonly used in stroke and neurology patients, and are not developed for this study [12, 15–17].

**Table 1:**
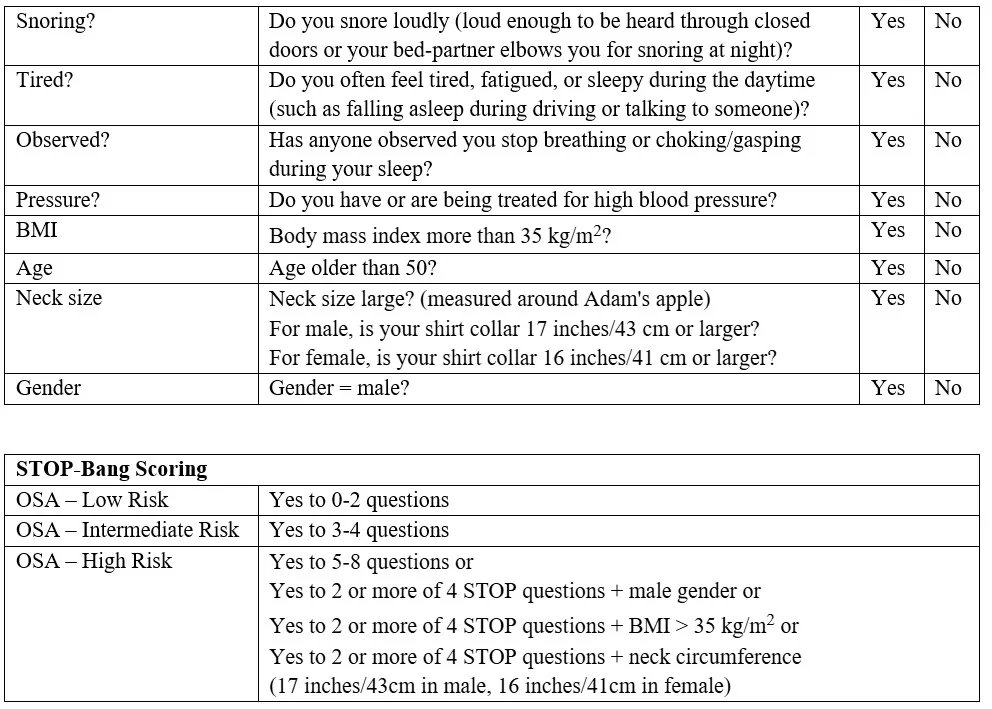
STOP-Bang questionnaire [18]

### Stroke etiology

Stroke etiology was assessed for all included patients based on information documented in discharge summaries and electronic medical records. Etiology was classified according to the Trial of Org 10172 in Acute Stroke Treatment (TOAST) criteria, which comprise five subtypes: large-artery atherosclerosis, cardioembolism, small-vessel occlusion, stroke of other determined etiology, and stroke of undetermined etiology.

In cases where the etiology was initially uncertain, classification was reassessed by a senior stroke physician using all available clinical, radiological, and diagnostic information, and patients were subsequently assigned to the most appropriate TOAST category.

### Pre-stroke risk factors

Hypertension and hypercholesterolemia were defined as ongoing treatment with antihypertensive or lipid-lowering medication prior to hospital admission. Diabetes mellitus was defined as a documented diagnosis prior to admission. Atrial fibrillation was considered present if previously diagnosed and documented in the medical records, including prior electrocardiogram (ECG) or telemetry recordings.

Only conditions known prior to admission were included in the main analysis. Patients in whom these conditions were newly diagnosed during hospitalization were not classified as having pre-existing risk factors in the baseline table. Consequently, the prevalence of vascular risk factors reported at baseline may underestimate the true burden of undiagnosed disease in the study population.

### Statistical analyses

All statistical analyses were performed using Stata/MP version 18.5 (StataCorp, College Station, TX, USA). Categorical variables are presented as numbers and percentages. Continuous variables are reported as mean ± standard deviation (SD) when normally distributed and as median with interquartile range (IQR) when skewed.

The chi-square test was used to assess both the association between stroke etiology and STOP-Bang risk categories, and between categorized ESS levels and STOP-Bang risk groups. The latter analysis was performed to see if there were significant differences between clinically relevant categorizations of ESS levels and STOP-Bang risk groups. Pearson’s correlation coefficient was used to assess the relationship between continuous ESS and STOP-Bang scores.

Multiple linear regression analyses were performed with continuous ESS score as the dependent variable and continuous STOP-Bang score as the primary independent variable. The model was adjusted for predefined potential confounders, including high coffee consumption, prior ischemic stroke, known neurological disease, known psychiatric disease and mRS score prior to admission. Continuous ESS and STOP-Bang scores were used in the regression analyses to evaluate associations across the full score range and to avoid additional categorization. Regression coefficients (β) with 95% confidence intervals (CI) are reported.

Statistical significance was defined as a two-sided p-value < 0.05. Model assumptions, including linearity and normality of residuals, were evaluated by inspection of residual plots.

## Results

### Study population

Of 140 screened patients, 16 were excluded for the following reasons: lack of confirmed stroke or TIA on CT or MRI (*n* = 8), incomplete questionnaire data (*n* = 4), premorbid mRS score ≥ 3 (*n* = 3), and prior intracerebral hemorrhage (*n* = 1). A total of 124 patients were included in the final analysis.

### Sociodemographic characteristics and risk factors

The sociodemographic characteristics and vascular risk factors of the study population are presented in Table 2. The mean age was 72.1 years (SD 12.5), and men constituted 57.3% of the cohort. Most participants were retired (68.6%), while 25.8% were currently working. Among potential confounders for daytime sleepiness, high coffee consumption (> 3 cups per day) was reported by 41.1% of participants. No patients reported to have a documented sleep disorder. Among vascular risk factors, diabetes mellitus was significantly associated with higher STOP-Bang scores (*p* = 0.034), whereas hypercholesterolemia, atrial fibrillation, smoking, and alcohol consumption were not significantly associated.

**Table 2:** Sociodemographic and risk factor characteristics (*n* = 124)

Variable	Value
Sociodemographic variables	
Sex	
* Men*	71 (57.3%)
* Women*	53 (42.7%)
Employment status	
* Working*	32 (25.8%)
* Not able to work*	7 (5.6%)
* Retired*	85 (68.6%)
Age, years	72.1 (12.5)
Risk factors before admission	
Body mass index, kg/m²	26.1 (4.3)
Previous ischemic stroke/TIA	31 (25.0%)
Medically treated hypertension	55 (44.4%)
Medically treated hypercholesterolemia	48 (38.7%)
Diabetes mellitus	21 (16.9%)
Active smoking (within the last month)	23 (18.6%)
Snus (tobacco)	9 (7.3%)
Atrial fibrillation	13 (10.5%)
Potential confounders for sleepiness	
Alcohol consumption (> 14 units/week)	6 (4.8%)
Coffee (> three cups/day)	51 (41.1%)
Sleep disorders	0 (0.0%)
Psychiatric disorders	11 (8.9%)
Neurological disorders	5 (4.0%)

Values are presented as mean (SD) or* n *(%)

### Stroke characteristics

Stroke characteristics of the study population are summarized in Table 3. Most patients were admitted with ischemic stroke (83.9%), while 16.1% were diagnosed with TIA. The median NIHSS score at admission was 2 (IQR 0–5). Patients with TIA were also assessed using the NIHSS, where patients with complete symptom resolution had an NIHSS score of 0 at discharge. Most patients did not receive acute-phase reperfusion therapy, with 72.6% undergoing neither thrombolysis nor thrombectomy. Large-artery atherosclerosis was the most common etiological subtype (44.4%), followed by cardioembolism (29.8%) and small-vessel disease (21.8%). No statistically significant difference in stroke etiology distribution was observed between STOP-Bang risk categories (χ² test, *p* = 0.82).

**Table 3:** Stroke characteristics (*n* = 124)

Variable	Value
Stroke characteristics	
Brain infarction	104 (83.9%)
TIA	20 (16.1%)
NIHSS	
* Admission*	2 (0–5)
* Day 1*	1 (0–3)
* Day 7/ discharge*	1 (0–3)
mRS	
* Pre-stroke*	1 (0–2)
* Day 1*	2.5 (1–4)
* Day 7/ discharge*	2 (1–3)
Brain infarction etiology	
* Large-artery atherosclerosis*	55 (44.4%)
* Cardioembolism*	37 (29.8%)
* Small-vessel occlusion*	27 (21.8%)
* Stroke of other determined etiology*	5 (4.0%)
Acute reperfusion treatment	
Thrombolysis	31 (25.0%)
Thrombectomy	3 (2.4%)
Neither thrombolysis nor thrombectomy	90 (72.6%)

Values are presented as median (IQR) or *n *(%)

### STOP-Bang score

**Fig. 1:**
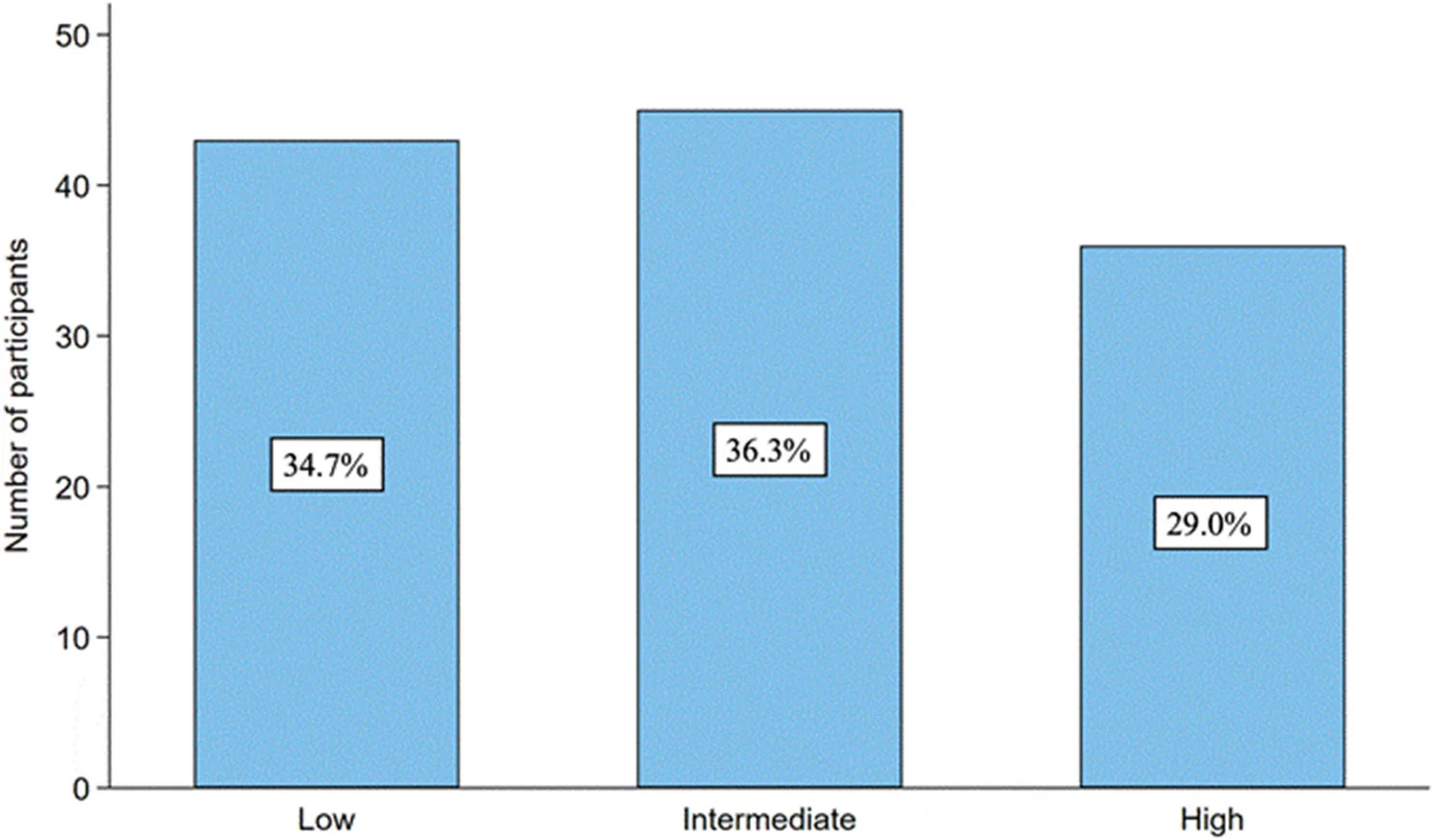
Risk categories for OSA based on the STOP-Bang questionnaire

The mean STOP-Bang score prior to admission was 3.2 (range 1–7). Based on the STOP-Bang questionnaire, patients were categorized into three risk groups for OSA. Overall, 65.3% of the study population was classified as having intermediate or high risk of OSA. The distribution of STOP-Bang risk categories is shown in Fig. 1.

The distribution of individual STOP-Bang components was as follows: snoring (*n* = 70), severe tiredness (*n* = 30), observed apnea (*n* = 24), known hypertension (*n* = 59), BMI > 35 kg/m² (*n* = 4), age > 50 years (*n* = 114), neck circumference > 40 cm (*n* = 22), and male sex (*n* = 71).

### Epworth sleepiness scale (ESS)

**Fig. 2:**
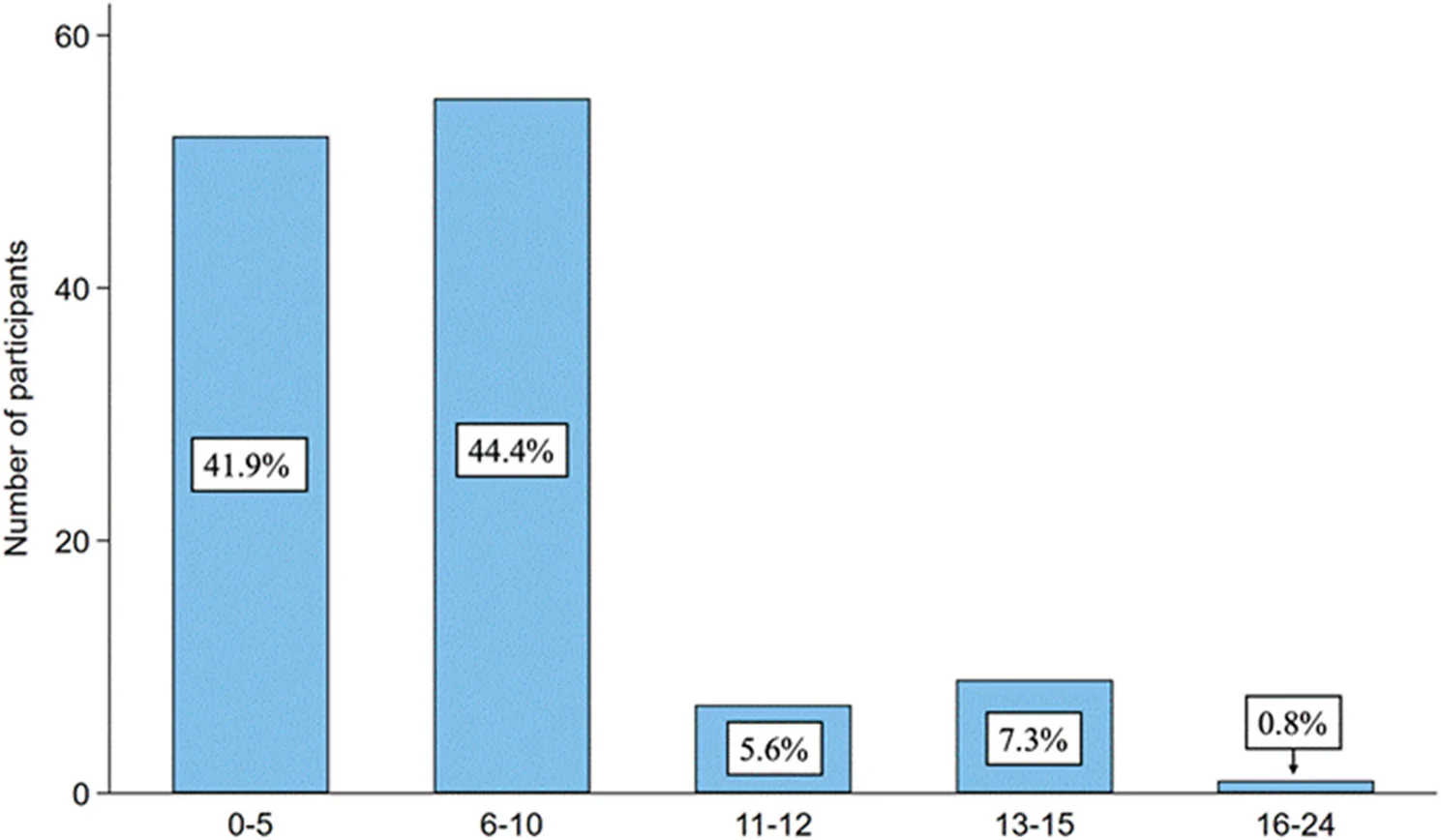
Categories of daytime sleepiness based on the ESS questionnaire. Patients were classified into five categories of daytime sleepiness: lower normal (0–5), higher normal (6–10), mild excessive (11-12), moderate excessive 13-15), and severe excessive daytime sleepiness (16-24)

The mean ESS score prior to admission was 6.5 (range 0–18). Overall, 17 individuals (13.7%) met the criteria for excessive daytime sleepiness, defined as an ESS score ≥ 11. The distribution of ESS categories is illustrated in Fig. 2.

### Association between STOP-Bang and ESS

Excessive daytime sleepiness (ESS ≥ 11) was observed in 13 of 81 patients (16.0%) with intermediate or high STOP-Bang risk, compared with 4 of 43 patients (9.3%) in the low-risk group (Table 4). No statistically significant association was observed between categorized STOP-Bang risk groups and ESS categories (χ² test, *p* = 0.30).

**Table 4:** Chi-square test of STOP-BANG severity and ESS

ESS score		Normal daytime sleepiness (0–10)	Excessive daytime sleepiness (11–24)	Total
STOP-BANGseverity	Low	39	4	43
	Intermediate/high	68	13	81
Total		107	17	124

Values are presented as number of participants (n). Excessive daytime sleepiness was defined as an ESS score ≥ 11

The correlation between the continuous ESS score and STOP-Bang score was 0.23 (*p* = 0.01). In regression analyses adjusting for predefined potential confounders, STOP-Bang scores were significantly associated with higher ESS scores (β = 0.67, 95% CI 0.22–1.12, *p* = 0.004). Among the covariates included in the model, only known psychiatric disease was independently associated with ESS (*p* = 0.004).

Further, we constructed a modified STOP-Bang score excluding vascular components (hypertension, BMI, age, and sex) and re-ran the same multivariable linear regression model with ESS as the dependent variable and adjustment for the same predefined covariates. The modified score remained significantly associated with ESS (β = 1.02, 95% CI 0.41–1.63, *p* = 0.001).

## Discussion

In the otherwise healthy general population, the prevalence of moderate to severe OSA—defined as an apnea-hypopnea index ≥ 15 events per hour—is estimated to be approximately 10–20% [12, 21]. In our study, 65.3% of participants were classified as having an intermediate or high risk of OSA according to the STOP-Bang questionnaire, indicating that a considerable proportion of patients hospitalized with acute ischemic stroke or TIA may have previously unrecognized sleep-disordered breathing. This is consistent with previous research where reported prevalences range from approximately 55% to 77%, depending on the screening method and diagnostic criteria used, demonstrating that OSA is common among individuals with cerebrovascular disease [2, 10, 13, 19, 20]. Additionally, it is estimated that 80% of individuals with moderate to severe OSA remain undiagnosed and untreated [27]. This background highlights the broader underdiagnosis of OSA and suggests that the true burden of disease may be higher than suggested by questionnaire-based estimates of OSA risk. However, the true prevalence of OSA in our cohort cannot be determined without objective sleep testing.

Direct comparisons between studies should be interpreted cautiously. Participants in the present study were considerably older, with a mean age of 72.1 years, compared with 47.8 years in the study by Hrubos et al. [21]. Furthermore, our cohort consisted exclusively of patients with ischemic stroke or TIA. These demographic and clinical differences may contribute to the higher observed risk of OSA.

Taken together, our findings support the growing body of evidence suggesting that OSA may be underdiagnosed among patients with ischemic stroke or TIA. Incorporating simple screening tools such as the STOP-Bang questionnaire into routine clinical assessment may therefore help identify patients who could benefit from further diagnostic evaluation for sleep-disordered breathing.

Excessive daytime sleepiness was present in 13.7% of participants. This proportion is lower than that reported by Jaussent et al. [22], who observed excessive daytime sleepiness in 33% of individuals in a large general population cohort, and in 27.7% of individuals aged over 58 years. Others estimate the excessive daytime sleepiness prevalence in the general population to range between 9% and 28% [23–26], while among patients with stroke estimates vary from approximately 6% to nearly 50% depending on assessment methods and patient characteristics [28]. The relatively modest prevalence observed in our study may reflect the limited sample size, the reliance on questionnaire-based assessment, and the characteristics of the study population, including higher age, low mRS, and the exclusion of patients with aphasia, cognitive impairment, brain hemorrhage, or known OSA. These selection criteria probably excluded a substantial proportion of the general population with stroke experiencing excessive daytime sleepiness beforehand.

Previous studies have found that the STOP-Bang questionnaire is more effective than ESS in identifying individuals at moderate-to-high and high risk of OSA [29, 30]. In our study, no association was observed between high STOP-Bang scores and ESS severity using chi-square testing. However, regression analysis adjusted for relevant covariates revealed a statistically significant association between continuous STOP-Bang and ESS scores (*p* = 0.004). This finding suggests that although subjective daytime sleepiness does not necessarily correspond directly to OSA risk categories, there may still be an underlying relationship between these measures when potential confounding factors are taken into account.

Stroke etiology in our cohort was assigned retrospectively after completion of the diagnostic evaluation. Such an approach is frequently applied in stroke research to allow etiological classification based on the complete set of clinical information, including vascular imaging, cardiac investigations, and prolonged rhythm monitoring. Early etiological classification during the acute admission may lead to a higher proportion of strokes being categorized as undetermined due to incomplete investigations. Centralized or retrospective adjudication after full diagnostic work-up has therefore been widely used in stroke cohorts to improve classification reliability and reduce the proportion of cryptogenic stroke. Accurate etiological classification is particularly important in studies examining potential mechanistic associations, such as OSA and stroke subtype, as misclassification of stroke etiology could obscure subtype-specific relationships.

Although large-artery atherosclerosis was the most common etiological subtype in our cohort, we did not observe an association between stroke etiology and STOP-Bang risk categories. This finding should be interpreted cautiously, as the study was not designed or powered to investigate mechanistic associations between OSA risk and stroke subtype. Nevertheless, previous studies support biologically plausible vascular and metabolic links between OSA and ischemic stroke, suggesting that cardiometabolic and vascular pathways may contribute to stroke risk in patients with OSA [31, 32], although such mechanisms could not be evaluated in the present study.

Stroke severity in our cohort was generally mild. The median NIHSS score at admission was 2. This is comparable to data from Norwegian Stroke Register, which reported a median NIHSS score of 1.3 among all ischemic stroke patients in Norway in 2024 [33]. Although our study population was relatively small, this similarity suggests that the cohort may reasonably reflect the local stroke population.

### Strengths and limitations

The observational design is well suited for estimating prevalence within defined populations. A strength of the present study is that assessments were performed during the acute phase of stroke. Mapping risk factors shortly after the incident reflects pre-stroke risk factors more accurately than later in the course of the disease. Previous studies have conducted interviews and testing to assess present OSA symptoms [2, 3, 13, 20], and most of them were conducted after the acute phase, whereas we interviewed patients in the acute phase about OSA symptoms prior to the incident. To our knowledge, our study is the first to take this approach.

The study included only patients with a mRS score ≤ 2, thereby focusing on individuals with mild to moderate disability. This criterion was intended to reduce potential confounding from severe neurological impairment or comorbid conditions that might independently influence sleep-disordered breathing, and to identify the population that may benefit most from primary stroke prophylaxis. However, the selection criteria may limit the external validity and generalizability of the findings to the broader stroke population, particularly patients with more severe strokes, pre-existing disability, institutionalized patients, or substantial cognitive and communication impairments. Since OSA and excessive daytime sleepiness may be even more prevalent in these groups, the true burden of sleep-disordered breathing in the overall stroke population could potentially be underestimated in the present study. While the sample size of 124 patients provided a reasonable basis for estimating prevalence, it may have limited the statistical power to identify subtle associations and explore differences across subgroups.

Another limitation concerns the identification of OSA. Although STOP-Bang is considered a reliable tool for identifying OSA, our study did not include objective confirmation with polysomnography, which remains the diagnostic gold standard [34]. Self-reported measures may introduce misclassification bias, as patients may underreport, overestimate, or fail to recognize symptoms related to sleep-disordered breathing. In addition, following a stroke, patients may over time have difficulty accurately recalling or interpreting relevant symptoms, which may further affect the reliability of questionnaire responses. However, the study sought to minimize recall bias by conducting interviews during the acute phase when the patients were likely to recall the incident and recent events more accurately.

Although polysomnography would provide objective diagnostic confirmation, its use in the acute phase after stroke presents practical and methodological challenges. Acute physiological disturbances may influence sleep architecture and respiratory parameters, potentially increasing the likelihood of false-positive findings. Furthermore, when sleep studies are conducted after the stroke event, the stroke itself may contribute to the development or worsening of sleep-disordered breathing, complicating causal interpretation.

A potential limitation of the STOP-Bang questionnaire is that several of its components overlap with established vascular risk factors for stroke, including hypertension, obesity and older age. This raises the possibility that STOP-Bang may partly reflect a general vascular risk profile rather than OSA-specific risk. In our cohort, this overlap was particularly evident for age, as 114 patients (92%) were older than 50 years, consistent with the demographic characteristics of the stroke population of interest. Male sex contributed less in this respect, as the sex distribution in the study population was more balanced, with 57.3% of the patients being male. Nearly half of the patients had hypertension, while only 4 patients had BMI > 35 kg/m², suggesting that not all vascular components contributed to the overall high STOP-Bang score. At the same time, 89 patients (71.8%) reported at least one of the four symptoms regarding snoring, severe tiredness, observed apnea and enlarged neck circumference, of whom 40 patients (32.3%) reported two or more of these non-vascular symptoms. Taken together, these findings indicate that the observed high STOP-Bang scores were not solely driven by vascular risk factors but also reflected a substantial burden of undiagnosed OSA-related symptoms in the stroke population. The modified multivariable linear regression model excluding vascular components was consistent with the primary analysis using the full STOP-Bang score, supporting the interpretation that the association between STOP-Bang and ESS was not solely driven by shared vascular risk factors. However, as this modified score is not a validated screening instrument, these findings should be interpreted with caution.

## Conclusions

In this study of patients with acute ischemic stroke or transient ischemic attack, a substantial proportion were at increased pre-event risk of previously unrecognized obstructive sleep apnea, fulfilling the primary aim of demonstrating a high burden of OSA-related symptoms prior to the cerebrovascular event. In contrast, excessive daytime sleepiness was relatively uncommon, indicating that subjective sleepiness alone is an insufficient marker of underlying sleep-disordered breathing in this population.

Addressing the secondary aims, the STOP-Bang questionnaire identified a markedly larger proportion of patients at risk of OSA than ESS identified with excessive daytime sleepiness. The proportion of patients at risk identified by the STOP-Bang questionnaire was consistent with previous studies reporting a high prevalence of OSA in stroke cohorts, supporting this questionnaire’s potential higher clinical and practical value for identifying patients at risk of OSA or patients who may benefit from further diagnostic evaluation for OSA. Although no significant association was observed between categorized STOP-Bang and ESS measures, regression analyses demonstrated a significant relationship between continuous scores, suggesting that the two instruments capture related but distinct dimensions of sleep-disordered breathing and sleep-related symptoms. This indicates that STOP-Bang and ESS may provide complementary information.

Overall, these findings support the notion that OSA is frequently underrecognized prior to stroke and TIA. Incorporating simple screening tools—particularly STOP-Bang—into routine clinical assessment may improve identification of patients who could benefit from further evaluation for sleep-disordered breathing, with potential implications for both primary and secondary stroke prevention.

## Data Availability

The datasets generated and/or analysed during the current study are not publicly available due to privacy restrictions but are available from the corresponding author on reasonable request and with permission from relevant authorities.

## Abbreviations

BMI: Body mass index
CI: Confidence interval
CPAP: Continuous positive airway pressure
CT: Computed tomography
ECG: Electrocardiogram
ESS: Epworth Sleepiness Scale
IQR: Interquartile range
MRI: Magnetic resonance imaging
mRS: Modified Rankin Scale
NIHSS: National Institutes of Health Stroke Scale
OSA: Obstructive sleep apnea
SD: Standard deviation
STOP-Bang: Snoring, Tiredness, Observed apnea, high blood pressure, Body mass index, Age, Neck circumference, Gender
TIA: Transient ischemic attack
TOAST: Trial of Org 10172 in Acute Stroke Treatment
